# Intimal Macrovascular Pericytes: Their Role in Vascular Biology and Atherogenesis

**DOI:** 10.2174/0109298673295675240826070754

**Published:** 2024-09-02

**Authors:** Alexander N. Orekhov, Elena R. Andreeva

**Affiliations:** 1 Institute of General Pathology and Pathophysiology, 8 Baltiyskaya Street, Moscow, 125315, Russia;; 2 Laboratory of Cell Physiology, Institute of Biomedical Problems, Russian Academy of Sciences, 76a Khoroshevskoye Shosse, Moscow, 123007, Russia

**Keywords:** Atherosclerosis, arterial wall, macrovascular pericytes, microvascular pericytes, smooth muscle cells, immune defense, lipid accumulation, extracellular matrix synthesis

## Abstract

Atherosclerosis remains a major challenge to global healthcare despite decades of research and constant trials of novel therapeutic approaches. One feature that makes atherosclerosis treatment so elusive is an insufficient understanding of its origins and the early stages of the pathological process, which limits our means of effective prevention of the disease. Macrovascular pericytes are cells with distinct shapes that are located in the arterial wall of larger vessels and are in many aspects similar to microvascular pericytes that maintain the functionality of small vessels and capillaries. This cell type combines the residual contractile function of smooth muscle cells with a distinct stellar shape that allows these cells to make numerous contacts between themselves and the adjacent endothelial layer. Moreover, pericytes can take part in the immune defense and are able to take up lipids in the course of atherosclerotic lesion development. In growing atherosclerotic plaques, the morphology and function of pericytes change dramatically due to phagocytic and synthetic phenotypes that are actively involved in lipid accumulation and extracellular matrix synthesis. In this review, we summarize our knowledge of this less-studied cell type and its role in atherosclerosis.

## 1 INTRODUCTION

Current consensus defines pericytes as a cell type that can be found in microvessels (capillaries, as well as pre-capillary arterioles and post-capillary veins), where they wrap around and form contacts with endothelial cells [1-5]. Pericytes are characterized by branched morphology (a stellate shape) that allows them to effectively form multiple contacts with other cells and the expression of contractile proteins like smooth muscular α-actin, which helps them regulate capillary blood flow through contraction [6-10]. The functions of pericytes remain fully defined, but they are known to be involved in microvascular flow regulation, serving as a source of progenitor cells and promoting angiogenesis. Moreover, their location immediately beneath the endothelial layer and multiple contacts with the endothelial cells make pericytes indispensable for endothelial maintenance [11-13]. Not surprisingly, this cell type has already been found to play a prominent role in health and disease, with a special impact on cancer and central nervous system disorders, where it participates in the maintenance of the blood-brain barrier [14-17]. However, detailed studies on the cell composition of the vascular wall have revealed that pericytes, or pericyte-like cells, are likely not to be restricted to microvessels but can be found in the intimal layer of macrovessels [18-20]. The initial stages of atherosclerotic lesion development take place in the innermost layers of the arterial wall: the endothelium and intima. Despite decades of active research, the processes that occur in these layers and the cell types involved remain to be fully characterized. It is clear that inflammatory activation and lipid accumulation in the arterial wall are key features of the early stages of lesion formation, and the inflammatory cells recruited from the bloodstream are actively involved in them. However, resident cells of the vascular wall also participate in this process.

In this review, we will focus on the cell type, which is similar to microvascular pericytes in terms of morphology, localization proximal to the endothelial cells, and antigen markers expression, but located in the intima of large vessels, which is immediately underlying the endothelial lining. We will term these cells macrovascular pericytes. Furthermore, we will summarize our knowledge of the morphological features and identification of these cells and their role in atherosclerosis development. For all clarity, capillary adventitial and vasa vasorum pericytes that can be found in complicated atherosclerotic plaques are beyond the scope of this review.

## 2 PERICYTE DISCOVERY AND CHARACTERIZATION

The first notion of pericyte-like cells in macrovessels was made more than 150 years ago when they sparked the interest of researchers with their unusual stellate shape, which was different from the elongated smooth muscular cells. They were first regarded as fibroblasts or fibrocytes [21]. Since their initial discovery by Langhans *et al.* in 1866, pericyte-like cells have been given different names. However, the provided descriptions of the cells’ morphology and multipotent features allowed assuming that the same cell type was already described: myointimal cells [22-26], multipotent and multifunctional mesenchymal cells [27-37], intermediate smooth muscle cells [38-40], intermediate cells [41-43], myoendothelial cells [44-47], modified smooth muscle cells [48-56], cambial cells of the subendothelium [57], intimacytes [58, 59], Langhans' cells [60], unidentified cells [61], primitive cells [62], or poorly differentiated cells [63]. During the era of light microscopy studies of arterial wall cell types, a consensus opinion was formed regarding the morphology and low level of differentiation of these cells. Studies of the mid-twentieth century classified these cells as fibroblasts [64], fibrocytes, histocytes, and monocytoid cells [65]. Some researchers regarded pericyte-like cells as an intermediate type between fibroblasts and smooth muscle cells [66]. Regarding the possible origins of these cells, their transformation from the endothelial cells was proposed, in which endothelial cells relocated into the space beneath the endothelial lining and took on functions typically unusual for the endothelium [12, 21-23]. Another suggestion was that these cells undergo division, after which they differentiate into endothelial or smooth muscle cells [10, 18]. It has also been proposed that stellate-shaped cells derived from pluripotent fibroblasts represent a pluripotent cell type that can differentiate into fibroblasts, endothelial or smooth muscle cells [22]. Such differentiation could take place in response to changing environments and specific cellular stimuli. Some researchers have suggested that, depending on the local environment and the organism's needs, pericyte-like cells can become fibroblasts, endothelial cells, or macrophages to participate in defense mechanisms. Hence, they should be regarded not as resting but as active pluripotent cell types [24]. The presence of low-differentiated cells in the intima of small arteries and veins was described as early as the mid-twentieth century [25].

Later studies that examined pericyte-like cells with increased resolution due to the employment of electron microscopy methods have described distinctive characteristics of these cells, including the presence of contractile filaments and their interaction with the vessel's basal membrane or its fragmented portions [26, 27]. These observations supported the earlier assumption that these cells were of smooth muscular origin [28]. Accordingly, later studies frequently termed these cells “modified smooth muscle cells,” which were different from regular smooth muscle cells by their degree of differentiation. It was proposed that smooth muscular cells populating the medial layer of the vascular wall could migrate into the intima and undergo modification, giving rise to the cells that change morphology but retain contractility. However, these cells could readily be distinguished from the smooth muscular cells by their stellate morphology, relatively poorly developed contractile apparatus, and active synthetic phenotype [13]. Alternative names, such as “contractile phenotype smooth muscular cells” and “synthetic phenotype smooth muscular cells,” were suggested and are still used. Attempts to define stellate cells as smooth muscle cells have received widespread support because of the concept of smooth muscle cells as a key cell type in atherogenesis. The important role of smooth muscle cells in the development of atherosclerotic lesions was highlighted by Russel Ross and his followers [44, 45, 67-76]. Later studies have demonstrated that the increase in stellate cells in the affected vascular intima was related to well-known features of atherosclerosis, such as intimal thickening, lipid accumulation, and increased collagen synthesis [77]. This potential importance to the disease development further spurred the researchers’ interest in the study of pericyte-like cells. Given their multipotent nature, diverse functions, and unique location, they could well be named the principal cell type involved in atherosclerosis development.

The smooth muscular origin of subendothelial pericytes remains the prevailing concept to the current day [78]. However, even early studies on stellate intimal cells have already pointed out that “most stellate cells differed sufficiently from typical smooth muscle cells to conclude that they represented another cell type in the intima” [79]. Some of the most recent studies have described macrovascular pericytes as distinct cell types based on their phenotype and marker expression [80].

## 3 IN SEARCH FOR MACROVASCULAR PERICYTES

Classification of macrovascular pericytes as smooth muscle cells can be justified by the historical context. For a long time, the prevailing theory of atherogenesis was that of Russel Ross and colleagues, who highlighted the key atherogenic role of smooth muscle cells, their migration to the growing lesion, and proliferation [73]. However, the possible role of other subendothelial cell types in atherogenesis and related processes was not properly considered. However, later works of other research groups, including our own, challenged this point of view, establishing the concept of stellate subendothelial cells being a distinct cell type, similar or identical to microvascular pericytes rather than smooth muscle cells (Table **1**) [77, 81-89].

Studies of our group helped describe the composition of subendothelial cells of healthy arterial walls and atherosclerotic lesions in more detail [89]. Immunocytochemistry with antibodies specific for antigens present in different cell types was used to characterize the subendothelial cell populations, including smooth muscle cells and other cell types (Table **2**).

Smooth muscle cells were revealed with the help of antibodies against smooth muscle α-actin. Cells positive for this antigen constituted around 50% of the total cell population both in normal tissue and in atherosclerotic lesions. Such cells normally had a round nucleus and two or more cytoplasmic processes. Pericytes were identified with specific antibodies against the 3G5 antigen. In atherosclerotic lesion sites, the proportion of cells positive for this antigen was lower, from 31% in the normal intima down to 5% in fibroatheromas (Table **3**) [90]. Most of these cells were in the subendothelial space of the intima while being very rare in the deeper muscular-elastic layer. Another pericyte antigen, 2A7, which is typical for pericytes in active angiogenesis sites, was present only in cells located in advanced atherosclerotic lesions, especially in lipofibrous plaques. Finally, the use of antigens typical for leukocytes and monocyte-macrophages helped distinguish the pericyte-like cells from these cell types, which were also present in atherosclerotic lesion areas. Interestingly, the use of an anti-CD68 antibody revealed not only typical macrophages (as assessed by the cell's overall morphology) but also cells with a distinct morphology, with multiple long processes. Double staining with anti-CD68 and anti-smooth muscle α-actin demonstrated that such unusual cells also contained actin filaments and were more abundantly present in atherosclerotic lesions. At the same time, staining with anti-MAC3, which is specific for macrophages and does not stain smooth muscle α-actin-positive cells, demonstrated that the number of typical macrophages was also increased in atherosclerotic lesions. Therefore, two populations of subendothelial intimal cells could be distinguished: resident cells that were positive for both pericyte and macrophage (CD68) antigens and blood-borne cells positive for typical monocyte-macrophage antigens that were recruited from the bloodstream.

Comparative quantitative analysis of both populations demonstrated that resident cells in the subendothelial layer of the arterial intima were more involved in the atherogenesis process.

An important step for the identification of stellate cells in the intima of large arteries was the detection of a continuum of periendothelial cells positive for the 3G5 pericyte antigen. Such cells were found beneath the subendothelial lining of the entire vascular bed from capillaries to the intima of large arteries and veins. In large vessels, cells positive for 3G5 were also revealed in the media and in the adventitia of vasa vasorum vessels. The continuous subendothelial network of these cells is maintained through intercellular contacts formed during the long cellular processes [87].

## 4 MACROVASCULAR PERICYTES FORMING 3-DIMENSIONAL CELLULAR NETWORK

**Fig. (1):**
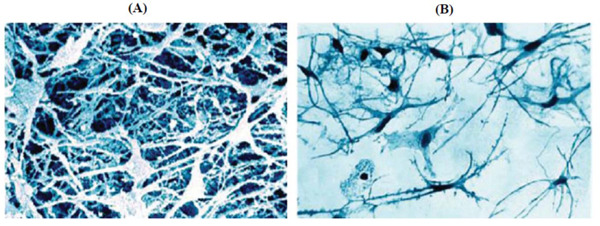
(**A**) Three-dimensional subendothelial network of stellate cells revealed by extracellular matrix dissociation (reproduced from [85]). (**B**) Suspension of human subendothelial intimal aorta cells after alcohol-alkaline dissociation [91].

The tissue organization of the subendothelial intimal layer of human arteries could be studied using the method of extracellular matrix dissociation. Treatment of tissue samples with enzymes that digest the extracellular matrix revealed the presence of a 3-dimensional cellular network located below the endothelial layer. This network was predominantly consisting of stellate cells (Fig. **1A**) [85]. Another approach is alkaline dissociation, in which tissue samples are treated with an alcohol-alkaline mixture. This method confirmed the presence of the subendothelial cellular network of stellate cells [91]. An example of a cell suspension after alcohol-alkaline dissociation of the subendothelial intima of a human aorta sample is shown in Fig. (**1B**). It can be seen that most of the cells have a stellate shape.

Within the network, cells are linked to each other horizontally and vertically through gap junctions [92].

## 5 THE ROLE OF MACROVASCULAR PERICYTES IN GROSSLY NORMAL INTIMA

The location and phenotypic features of macrovascular pericytes make them act as a second line of defense in the arterial wall [93]. The first defense line is represented by the endothelial lining. Local endothelial activation and increased permeability promote the recruitment of circulating immune cells. The second line, located immediately beneath the endothelial layer, can perform some functions typical for immune-competent cells, such as macrophages and dendritic cells. Indeed, pericytes can engage in phagocytosis and participate in cell signaling. Moreover, the earlier results indicated their involvement in antigen processing and presentation alongside dendritic cells [93].

Immunohistochemistry revealed the expression of HLA-DR (a major histocompatibility complex class II molecule) and CD3 (which is required for T-cell activation) in the areas of diffuse intimal thickening. The expression of HLA-DR positively and significantly correlated with the presence of lipid deposits, which were observed both intracellularly and extracellularly along the elastic fibers in the tissue. Moreover, double staining for these markers demonstrated that cells positive for HLA-DR were in direct contact with one another in the juxtaluminal sublayer of the arterial intima [94]. Examination of healthy aortic wall tissue for distribution of CD1a, which mediates antigen presentation of predominantly lipid and glycolipid antigens, also revealed the involvement of the subendothelial network of stellate cells. In this study, the use of “Hautchen” specimens (*en face* preparations of the tissue that allow examining thin layers of the tissue sample) showed that CD1a-positive cells were stellate in shape, with multiple long processes, and formed cellular networks in the subendothelial layer. Such cells represented approximately 10% of the total cellular population. The HLA-DR staining was most abundant in the middle third of the normal intima and less in the superficial and deeper layers, with CD1a-positive cells also being rare in the deeper layers of the arterial wall [95].

These observations allow the conclusion that macrovascular pericytes are likely to execute some of the functions of immune cells, representing a second line of defense within the arterial wall to respond to external pathogens or internal changes in the environment, including the presence of atherogenic LDL particles. Although the efficiency of immune defense processes performed by pericytes is not high compared with the specialized immune cells, their effectiveness is offset by their abundance in the normal arterial wall. Cell population analysis of the subendothelial intima revealed that stellate cells outnumber macrophages by 10 to 1 [90] and dendritic cells by 100 to 1 [96].

Numerous studies have indicated macrovascular pericytes of human arteries as one of the principal cell types involved in lipid accumulation in atherosclerosis. It indicates the high capacity for phagocytosis of these cells that can engulf relatively large lipid aggregates in the same manner as the specialized phagocytic cells, such as macrophages. In lipid-rich atherosclerotic lesions, pericytes represent the most abundant source of foam cells. These lipid-laden cells accumulate cholesterol through the phagocytic route, which leads to intracellular storage rather than rapid metabolism of the internalized lipids [77, 84, 85, 97].

**Fig. (2):**
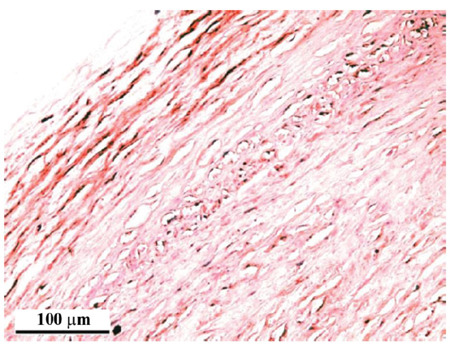
Immunohistochemical identification of TNF-α and muscle α-actin in tissue sections of human aorta. MultiVision Polymer Detection System has been used for simultaneously revealing two proteins. TNF-α is stained by chromogen LVBlue in blue color; α-actin is stained by chromogen LVRed in red color. Both stains are visible in close proximity [98].

Besides active phagocytosis, pericytes share other functions of macrophages, taking part in pro-inflammatory signaling and innate immune response. Immunohistochemical analysis demonstrated the expression of TNF-α in cells positive for smooth muscle α-actin in the human aortic wall (Fig. **2**) [98]. Moreover, the expression of both TNF-α and CCL18 (C-C motif chemokine ligand 18) was up-regulated by exposure of cells to atherogenic-modified LDL [98].

The role of pericytes in the inflammatory response could be further clarified in *in vitro* studies that used cultured cells of different origins. It was found that Mesenchymal Stromal Cells (MSCs) that are ontogenically related and phenotypically close to pericytes and human brain vascular pericytes demonstrated stimulation with lipopolysaccharide, a well-known pathogen-associated molecular pattern sensed by toll-like receptor 4, altered the expression patterns in these cells. In particular, the expression of chemokines and cytokines (10 genes in total) and adhesion molecules (ICAM1, VCAM1, and SELE) was increased by more than 5 folds. The authors have demonstrated that this up-regulation was NF-κB-dependent [99]. A study on microvascular pericytes *in vivo* reported that these cells coordinate interstitial migration of leukocytes in response to the inflammatory stimuli through up-regulation of ICAM-1 expression [100]. Stimulation of microvascular pericytes with IL-17 was found to induce granulocyte colony-stimulating factor and granulocyte-macrophage colony-stimulating factor production, thus prolonging neutrophil survival. Interestingly, IL-17-stimulated pericytes also enhanced neutrophils’ phagocytic capacity and induced neutrophil synthesis of IL-1α, IL-1β, TNF-α, macrophage migration-inhibitory factor, and IL-8. Therefore, pericyte secretome could attract diverse professional immune cells to the inflammation site [101].

The pro-inflammatory response of macrovascular pericytes can be induced by interaction with modified low-density lipoprotein (LDL). When naturally occurring, multiply modified LDL isolated from the blood of atherosclerotic patients was added to the primary cell culture, accumulation of the main classes of intracellular lipids and an increase in the secretion of inflammatory molecules were observed (Table **4**) [93]. At the same time, native LDL isolated from the blood of healthy individuals did not exert such an effect.

Antigen presentation properties of vascular cells have become an interesting research topic recently, but distinguishing the role of macrovascular pericytes in this process is challenging due to the limitations of the models using cultured cells from different species and different areas of the vascular wall [102]. Nevertheless, vascular smooth muscular cells from the large vessels were found to express low levels of class I MHC and up-regulate the expression of both class I and class II MHC in response to IFN-γ. Both vascular smooth muscle cells and microvascular pericytes were reported to express indolamine 2,3-dioxygenase (IDO), which contributed to T-cell inhibition. Furthermore, microvascular pericytes expressed PD-L1 and PD-L2 (negative co-stimulatory receptors) in response to IFN-γ stimulation. A more recent study showed that unstimulated microvascular pericytes could directly present alloantigen to TEM (effector memory T cells), while IFN-γ-activated pericytes instead suppressed TEM proliferation, but not cytokine production or signaling. Stimulation of pericytes with IFN-γ induced up-regulation of the IDO1 gene in comparison to IFN-γ-treated endothelial cells. It was suggested that immunosuppressive properties of human pericytes resulted from IFN-γ-induced IDO1-mediated tryptophan depletion [103]. These observations indicated that arterial wall cells are capable of antigen presentation by dampening the T- cell response stimulated by the activation of the endothelial cells.

## 6 MACROVASCULAR PERICYTES IN ATHEROGENESIS

The main features of the atherosclerotic process in the arterial wall are lipidosis, which is intra- and extracellular lipid accumulation, hypercellularity because of increased proliferation of the resident cells, as well as enhanced migration of circulating cells, and fibrosis resulting from locally increased synthesis of the extracellular matrix. Histologic studies have demonstrated that these processes are associated with an increase in the number of stellate cells (macrovascular pericytes) in the affected areas [77]. Considering the close involvement of pericytes in the main processes of atherogenesis, it can be hypothesized that this cell type plays a crucial role in the initial stages of atherosclerotic plaque development [104]. This role of macrovascular pericytes is described in more detail below.

Lipidosis is one of the early signs of atherosclerotic lesion formation, starting as early as the initial lesion stage. The study of cell populations isolated from unaffected aortic intima and atherosclerotic lesions of different stages by means of alcohol-alkaline dissociation allowed the evaluation of intracellular lipid accumulation manifested as the presence of intracellular lipid inclusions. It was found that cells containing lipid inclusions were present mostly in the subendothelial intimal and partly in the media-adjusted hyperelastic layer of the arterial intima. In the grossly normal intima and fatty streaks, 15% and 25% of the extracted cells, respectively, contained visible lipid inclusions. In developed atherosclerotic plaques, around 10% of cells contained lipid inclusions, and these cells localized in the hyperelastic layer. Interestingly, among the isolated cells, those containing lipid inclusions belonged predominantly to the subpopulation of stellate cells (macrovascular pericytes), accounting for 5-35% of cells [85].

The structure of the cellular network appears to be changing in the course of atherosclerotic lesion development. In the normal intima, the network contains cells of varying shapes that have probably also varying origins. Immediately below the endothelial layer, the network consists mostly of stellate cells, while in the deeper layers of the arterial wall, more elongated, spindle-like cells can be observed. A study on aortic areas affected by atherosclerosis, where fatty streaks were present, demonstrated that such areas were characterized by impaired cellular contacts and the presence of lipid droplets. Fatty streak areas also contained monocyte-like cells that had a rounded shape. Both the round and stellate cells contained lipid inclusions, suggesting intracellular lipid accumulation. Interestingly, cellular contacts appeared to be preserved on the margins of an atherosclerotic lesion but destroyed in the central areas of the plaque that contained giant stellate cells embedded in the connective tissue matrix. The process of cellular network disintegration was already visible in fatty streaks, where thinning and arborization of contact-forming processes of the stellate cells were observed. It is reasonable to assume that the loss of intercellular contacts in the network can play a role in the development of atherosclerotic plaques [85]. A more detailed study on the loss of intercellular contacts in the course of atherosclerotic development was performed *in vitro*. Incubation of cultured human aortic subendothelial cells with LDL immobilized on latex beads, LDL-free control beads, or atherogenic LDL modified through desialylation resulted in the alteration of intercellular contacts that resembled that occurring in the fatty streaks. It is possible that the presence of large particles and/or lipid aggregates that stimulate phagocytosis may lead to a change in the cellular phenotype towards a phagocytic one and result in intracellular lipid accumulation and loss of intercellular contact formation [86].

Intercellular communication through gap junctions can be studied *in vitro* using the fluorescent dye transfer technique. Studies on cell cultures of different origins demonstrated that communication was higher in cultures of SMCs isolated from grossly normal intima as compared to cultures of less differentiated cells, such as skin fibroblasts, fetal fibroblasts, or fetal aortic cells [92]. Interestingly, lipid-laden subendothelial intimal cells from atherosclerotic lesions showed reduced ability to intercellular communication, which was as much as 2.4 times lower than in cells from unaffected areas. The observed impaired communication is likely reflecting reduced differentiation of subendothelial cells of human arterial intima associated with atherosclerotic lesion development.

**Fig. (3):**
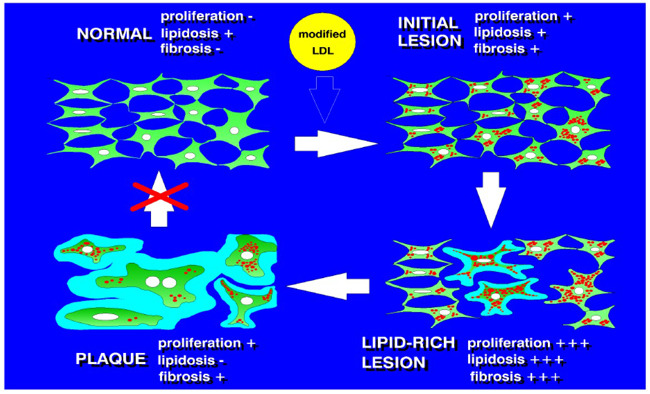
A scheme of participation of pericytes in atherogenesis.

The expression of connexin 43 and the presence of connexin plaques can be another marker of intercellular contact formation. A study on aortic tissue samples and cultured subendothelial cells demonstrated that connexin 43 expression and the number of gap junctions formed by connexins were significantly reduced in the arterial intima affected by atherosclerosis [97]. Moreover, exposure to atherogenic-modified LDL was found to reduce intercellular communication through gap junctions, therefore confirming the possible role of intracellular lipid accumulation and foam cell formation in the disintegration of the cellular network (Fig. **3**) [97].

Modelling atherosclerosis-related processes as proliferation (measured by means of DNA synthesis assessment), fibrosis (reflected by protein synthesis), and lipidosis (intracellular cholesterol accumulation) allowed demonstrating a correlation with the degree of contact formation between subendothelial cells positive for smooth muscle α-actin [97]. In cultured cells, the relationship between contact formation and proliferation and fibrosis was bell-shaped, with increased proliferation and protein synthesis at the initial stages of contact formation, subsequent suppression of both processes and a further increase in contact communication between the cells. This phenomenon has been referred to as “contact inhibition”. Studies on different stages of atherosclerotic lesions demonstrated the apparent bell-shaped distribution of intensity of proliferation, collagen synthesis, and lipidosis along the lesion evolution from the initial lesion and fatty streak to lipofibrous and fibrous plaques. In fibrous plaques, these processes appeared to be suppressed to a level lower than in unaffected intima. At the same time, the intensity of cellular communication decreased steadily across the lesion development stages, reaching its minimum in the fibrous plaques [97]. Atherosclerotic plaque development is driven by both the proliferation of residents and the infiltration and proliferation of circulating blood-borne cells. A detailed study on the cellular composition of atherosclerotic lesions from human coronary and carotid arteries demonstrated that blood-borne cells accounted for 1/3 to 1/2 of the total cell population, while lesions from the aorta contained not more than 15% of such cells. In the coronary and carotid arteries, the proliferation of cells visualized by PCNA staining revealed a similar bell-shaped relationship between the number of proliferating cells and the stage of lesion development from initial lesions to fibrous plaques [105].

## 7 ORIGINS OF MACROVASCULAR PERICYTES

The origins of macrovascular pericytes and their ontogenic relationship with microvascular pericytes remain to be a matter of debate. Most of the published studies on pericyte cell biology focus on brain pericytes or microvascular pericytes that have well-established sets of identification markers and recognized roles in the pathogenesis of actively studied human disorders. Macrovascular pericytes are often not distinguished from other smooth muscle α-actin-expressing cells (vascular smooth muscle cells). Careful studies on the large vessels’ walls, however, allow the identification of cells that are distinct from regular vascular smooth muscle cells by their location, morphology, and, probably, function [80]. However, the key to describing the macrovascular pericyte population and its origins might be considering their multipotent properties. Indeed, microvascular pericytes express a range of markers that are shared with Mesenchymal Stem Cells (MSCs), a population of multipotent adult stem cells that can be isolated from the progenitor cells present in the vascular wall. It has been demonstrated that MSC cultures could be obtained from microvascular pericytes and adventitial progenitor cells isolated from the walls of large arteries that also express MSC surface markers *in situ* [106].

The presence of common antigens in MSCs, microvascular pericytes, and macrovascular progenitor cells may indicate that MSCs may originate from pericytes through dedifferentiation or that MSCs may serve as progenitors for pericytes and pericyte-like cells. There is, however, a third possibility that a common progenitor for MSCs and pericytes exists in the vascular wall. In fact, various populations of progenitor cells could be isolated from intima, media, and adventitia of blood vessels and stimulated to differentiate into smooth muscular cells or have osteo-, chondro-, and adipo-genic potential. Interestingly, a population of multipotent vascular stem cells was identified, which could be differentiated into MSC-like cells and subsequently into smooth muscular cells and might play an important role in vessel remodeling in response to injury [107]. It is also possible, however, that the abovementioned studies were dealing with the same cell type, namely, pericytes, which are characterized by considerable phenotypic plasticity and can adapt to the surrounding milieu and stimuli. Hence, the contractile function may be of importance for microvascular pericytes that take part in maintaining the microvessel tonus. By contrast, proliferative and synthetic phenotypes can develop in response to stimuli, such as vessel damage or the presence of certain factors, such as circulating atherogenic LDL. Improving cell biology and microscopy methods will help clarify the origins and plasticity of this interesting cell type, which is likely to play a crucial role in the development of vascular diseases.

## CONCLUSION

Macrovascular pericytes that form the subendothelial three-dimensional network can be regarded as a key cellular type of the arterial wall of large arteries in healthy and diseased states. These cells serve as the second line of defense in the arterial wall after the endothelial lining and react to changing environmental factors alongside the specialized immune cells. In atherosclerosis, the pericyte network gradually disintegrates as a result of lipid uptake and accumulation by pericytes. This is accompanied by increased cellularity, the consequence of increased proliferation and fibrosis, and the result of enhanced extracellular matrix synthesis. Lipidosis, hypercellularity, and fibrosis are the main features of atherosclerosis plaque development, and macrovascular pericytes appear to play a key role in these processes. Macrovascular pericytes are likely to originate from capillary pericytes, but the exact origin of these cells remains to be determined.

## AUTHORS’ CONTRIBUTIONS

It is hereby acknowledged that both authors have accepted responsibility for the manuscript's content and consented to its submission. They have meticulously reviewed all results and unanimously approved the final version of the manuscript.

## Figures and Tables

**Table 1:** Comparison of features of microvascular pericytes, stellate pericyte-like cells, and smooth muscle cells.

**Feature**	**Reference**	**Cell Type**		
		**Microvascular Pericytes**	**Pericyte-like Cells**	**Smooth Muscle Cells**
Subendothelial localization	[77, 82-86]	+	+	-
Dense population in the tissue	Evidently	-	-	+
Stellate shape	[77, 82-86]	+	+	-
Smooth muscle α-actin	[87]	+	+	+
O-sialoganglioside (3G5)	[88, 89]	+	+	-
High molecular weight melanoma- associated antigen (2A7)	[88, 89]	+	+	-
Sialomucin (CD68)	[81]	+	+	-
Synthetic organelles	[77]	+	+	-+
Contractile structures	[77]	+-	+-	+

**Table 2:** Antibodies used for immunocytochemistry analysis of the arterial wall cellular composition.

**Antigen**	**Description**
2B11	Common leukocyte antigen
PD7/26 (CDLC)	
TÜK4 (CD14)	Monocyte-macrophage differentiation antigen
CDLC+CD14	Combination revealing blood-borne cells
CD68	Macrophage antigen
3G5	O-syaloganglioside
2A7	High molecular weight melanoma-associated antigen
aSM-1	Anti-smooth muscle α-actin

**Table 3:** Immunocytochemistry characterization of intimal cells of human aorta.

**Lesion Type**	**The Proportion of Positively Stained Cells, % of the Total Number of Cells** **(in Brackets the Number of Samples Analyzed)**				
	**Smooth Muscle α-actin**	**CDLC**	**CD68**	**3G5**	**2A7**
Normal (0)	47.6+2.3 (4)	2.2+0.4 (3)	3.9+0.4 (5)	31.3+7.0 (4)	0.0+0.0 (3)
Initial lesion (I)	47.2+3.1 (3)	6.2+1.2 (4)	6.1+1.4 (4)	6.3+1.0* (3)	1.2+0.3 (3)
Fatty streak (II)	42.2+3.1 (4)	5.0+0.9* (3)	13.2+0.8* (5)	11.7+2.0* (8)	3.0+0.7 (3)
Lipofibrous plaque (Va)	47.0+10.9 (5)	6.2+1.8* (9)	13.1+2.3* (4)	5.0+0.7* (5)	27.0+3.1 (3)

**Note:** * - Statistically significant difference from the number of positively stained cells in normal areas of human aortic intima, *p* < 0.05. Reproduced from [90].

**Table 4:** The effect of modified LDL on lipid accumulation and cytokine secretion in cultured human intimal cells.

	**Cytokine Secretion**			
	-	**TNF-α**	**IL-1**	**HLA-DR**
	**% of Control**			
Control	100±3	100±2	100±12	100±12
Native LDL	105±7	110±8	115±10	107±4
Modified LDL	201±27*	142±4*	156±4*	167±6*

**Note:** * - Statistically significant difference from the control (*p*<0.05).

## LIST OF ABBREVIATIONS

CCL18: C-C Motif Chemokine Ligand 18
DNA: Deoxyribonucleic Acid
HLA-DR: Human Leukocyte Antigen - DR Isotype
ICAM: Intercellular Adhesion Molecule
IDO: Indolamine 2,3-dioxygenase
IFN-γ: Interferon Gamma IL, Interleukin
LDL: Low-density Lipoprotein
MHC: Major Histocompatibility Complex
MSCs: Mesenchymal Stem Cells
PCNA: Proliferating Cell Nuclear Antigen
PD-L: Programmed Death-ligand
SELE: E-selectin
TEM: Effector Memory T Cells
TNF-α: Tumor Necrosis Factor-alpha
VCAM: Vascular Cell Adhesion Molecule
